# Transplantation of bone marrow-derived mesenchymal stem cells expressing elastin alleviates pelvic floor dysfunction

**DOI:** 10.1186/s13287-016-0308-1

**Published:** 2016-04-05

**Authors:** Minfei Jin, Ying Chen, Yun Zhou, Yan Mei, Wei Liu, Chenhao Pan, Xiaolin Hua

**Affiliations:** Department of Obstetrics and Gynecology, Xinhua Hospital, Shanghai Jiaotong University School of Medicine, Shanghai, 200092 China; Department of Orthopaedic Surgery, Shanghai Jiaotong University Affiliated Sixth People’s Hospital, Shanghai, 200233 China

**Keywords:** Pelvic floor dysfunction, Bone marrow-derived mesenchymal stem cells, Basic fibroblast growth factor, Stress urinary incontinence

## Abstract

**Background:**

Pelvic floor dysfunction (PFD) is a group of clinical conditions including stress urinary incontinence (SUI) and pelvic organ prolapse (POP). The abnormality of collagen and elastin metabolism in pelvic connective tissues is implicated in SUI and POP.

**Methods:**

To reconstitute the connective tissues with normal distribution of collagen and elastin, we transduced elastin to bone marrow-derived mesenchymal stem cells (BMSC). Elastin-expressing BMSCs were then differentiated to fibroblasts using bFGF, which produced collagen and elastin. To achieve the sustained release of bFGF, we formulated bFGF in poly (lactic-co-glycolic acid) (PLGA) nanoparticles (NP).

**Results:**

In an *in vitro* cell culture system of 7 days, when no additional bFGF was administrated, the initial PLGA-loaded bFGF NP induced prolonged production of collagen and elastin from elastin-expressing BMSCs. *In vivo*, co-injection of PLGA-loaded bFGF NP and elastin-expressing BMSCs into the PFD rats significantly improved the outcome of urodynamic tests. Together, these results provided an efficient model of connective tissue engineering using BMSC and injectable PLGA-loaded growth factors.

**Conclusions:**

Our results provided the first instance of a multidisciplinary approach, combining both stem cell and nanoparticle technologies, for the treatment of PFD.

## Background

Pelvic floor dysfunction (PFD) is the term for a group of clinical conditions, including stress urinary incontinence (SUI), pelvic organ prolapse (POP), overactive bladder syndrome, and fecal incontinence. SUI and POP are highly related to childbirth-associated pelvic floor injury [1]. Studies have estimated that 50 % of parous women have different degrees of SUI and POP. These disorders are prevalent in adult women and their incidences increase with age [2, 3]. Treatments for these conditions are still conservative and symptom based. Women with symptoms who have failed or declined conservative management are candidates for surgery. Traditionally, surgeries include anterior, posterior, or total repair of the vagina, with concomitant hysterectomy, but the recurrence rate can be as high as 20–30 % [4, 5]. Eleven percent of women over the age of 80 undergo surgery for such conditions, with an additional 30 % requiring repeated surgery [2, 3]. Synthetic and biomaterial meshes have been used recently to provide improved long-term outcomes during surgical treatments; however, about one-third of meshes cause scarring, erosion, and pain [6]. Alternative methods are therefore needed to promote the repair and regeneration of damage tissues.

All symptoms of PFD can be associated with pelvic floor defects. The pelvic floor support is provided by the interaction between the muscles and connective tissues within the pelvis, modulated by the neuro-pathway. Weakness in any of components without compensation will eventually increase the incidence of pelvic floor defects. Petros et al. [7] indicated that connective tissues, rather than muscle damage, were probably the major cause of SUI. Evidence suggests that abnormalities in connective tissue structure or its repair mechanism may be predispositions to prolapse [8]. Fibrous connective tissue is defined as the extracellular matrix (ECM), which mainly contains fibrous proteins including collagen and elastin produced by fibroblasts in the ECM. Fibroblasts also secrete growth factors that modulate the synthesis and breakdown of the fibers. Jackson et al. [9] reported that deficient collagen metabolism in the connective tissues may lead to PFD. Since then, the role of fibroblasts that produce both collagen and elastin has been studied widely. Both an increase [10–12] and a decrease [9, 13, 14] in the total content of collagen in connective tissues by different methods have been reported in PFD. Change in the ratio of subtypes of collagens and, more importantly, deficient crosslinking of collagens are involved in PFD [15–17]. Elastin, an insoluble polymer of the monomeric soluble precursor tropoelastin, is the main component of elastic fibers in the ECM in a variety of connective tissues including the aorta and ligaments. Elastin helps tissues to stretch and return to their original shape without energy input, which is extremely important in the regenerative system. Many studies have shown that elastin metabolism is affected in PFD because of increased degradation, abnormal synthesis, and interruption in elastin homeostasis [17–20].

Stem cells are able to participate in tissue repair due to their multilineage differentiation ability, potentially into various cell types of connective tissues, and therefore hold great promise for treating PFD. Bone marrow-derived mesenchymal stem cells (BMSCs) are one of the most well-characterized stem cell sources, have great differentiation capabilities, and secrete bioactive factors that benefit tissue repair [21]. In animal models of SUI, periurethral injection of BMSCs restored the damaged external urethral sphincter, and significantly alleviated SUI symptoms [22, 23].

Basic fibroblast growth factor (bFGF) [24], a member of the fibroblast growth factor family, is an important growth factor for mesenchymal stem cell proliferation [25], and is also required for the differentiation of BMSCs into fibroblasts [24]. However, bFGF has a short half-life, and therefore repeated injections are required to maintain stable local concentration, resulting in a high cost of relevant therapies. Better and more cost-effective methods are therefore needed to maintain the local concentration within the therapeutic duration.

Fibroblasts secrete collagen and elastin into connective tissues, with collagen being the predominant component. Elastic fibers play an important role in preventing collagen forming or remodeling into dense tissues. Contigen, a Food and Drug Administration (FDA)-approved injectable collagen solution, was discontinued in 2011 because its injection caused the formation of dense scar tissue comprising collagen without a significant amount of elastin, which contributed to a recovery rate as low as 15 % in 3 years [26]. Li et al. [27] reported that transduction of elastin to BMSCs could enhance the production of elastin, and transplantation of autologous BMSCs overexpressing elastin preserved the connective tissues with regional elasticity, preventing cardiac dilation and improving cardiac functions. Therefore, the goal of our current study is to reconstitute the connective tissues with normal distribution of collagen and elastin that would benefit the correction of pelvic support defects.

In the current study, we transduced primary cultured rat BMSCs with a high-efficiency adenoviral vector overexpressing elastin. To direct tissue repair and regeneration, the elastin-overexpressing BMSCs were induced to differentiate into fibroblasts under the unique culture condition, in which bFGF was encapsulated in poly(lactide-coglycolide-co-caprolactone) polymer nanoparticles (NPs)—the bFGF-loaded poly(lactic-co-glycolic acid) (PLGA) system. This system allows sustained release of bFGF into the culture. Injecting both the elastin-expressing BMSCs and bFGF-loaded PLGA NPs into the weakest position in the pelvis [28] of PFD rats has led to sustained release of bFGF, local differentiation of BMSCs to fibroblasts, as well as subsequent production of collagen and elastin. We hereby demonstrate that transplantation of elastin-expressing BMSCs improved conscious cytometry (CMG) and leak point pressure (LPP) in a rat PFD model. Our current study therefore provides a novel and multidisciplinary approach, combining both stem cell and NP techniques, to restore the pelvic floor supportive function.

## Methods

### Primary rat BMSC isolation and culture

Six-month-old female Sprague Dawley rats were used in the study. All animal studies were conducted in accordance with the rules and regulations of the Institutional Animal Care and Use Committee at Xinhua Hospital, Shanghai Jiaotong University School of Medicine, Shanghai, China. This study was approved by the ethics committee at Xinhua Hospital. Femurs from the rats were dislocated under anesthesia. Bone marrow cells were flushed from the femurs using 10 ml syringes containing 5–10 ml cold Iscove’s modified Dulbecco medium (IMDM). The cell suspension was centrifuged at 150 × *g* for 5 minutes, and the cell pellet was resuspended in IMDM. The cell suspension was layered onto Percol separation solution (density 1.073 g/ml, Sigma-Aldrich, St. Louis, MO, USA) and centrifuged at 400 × *g* for 30 minutes at 26 °C. The resulting cotton-like cells at the interface were collected and rinsed once. Cells were then cultured with IMDM supplemented with 20 % fetal bovine serum and 1 % streptomycin/penicillin (Sigma-Aldrich, St. Louis, MO, USA). The medium was changed after 24 hours and cells were maintained in the same medium until reaching approximately 80 % confluence. Cells were then trypsinized, replated, and cultured until the second passage. The yields of plastic-adherent, fibroblast-like cells from these two passages were obtained and considered the mesenchymal stem cells. These cells are harvested for characterization and used for the elastin virus infection.

### Transduction of BMSCs with elastin-expressing adenovirus vector

Transduction of BMSCs with elastin-expressing adenovirus vector was performed according to previously described methods [27]. The coding region of rat elastin was amplified by PCR. The PCR product was then cloned into a modified adenovirus (Ad) vector under control of the cytomegalovirus (CMV) promoter green fluorescent protein (GFP) (Ad-CMV-GFP). The final construct was referred to as Ad-CMV-GFP-elastin and confirmed by DNA sequencing. To generate the virus particle, AD-293 cells were transfected with Ad-CMV-GFP-elastin or empty vector Ad-CMV-GFP. Twenty-four hours after transfection, the medium was changed and transfected cells were cultured for 10–14 days. The supernatant (primary virus stock) was then harvested. The plaque forming units of the virus stock were measured by infecting AD-293 cells and counting the number of GFP-positive cells. The virus stock was also tested by PCR to confirm the presence of the elastin genes. To infect the BMSCs, the primary virus stock of Ad-CMV-GFP-elastin or Ad-CMV-GFP was added to BMSCs and, after incubation for 24 hours, medium containing the virus was replaced with complete culture medium and the cells were incubated for an additional 24 hours. The efficiency of infection was evaluated by the percentage of GFP-positive cells. Five days after infection, cells were harvested for evaluation of elastin expression by western blotting and PCR. No alteration of BMSC surface CD marker was observed by flow cytometry.

### Formulation of bFGF-loaded poly(lactide-coglycolide-co-caprolactone) polymer NPs

Formulation of PLGA-NPs was performed according to previously described methods [29, 30]. A double emulsion–solvent evaporation method (water–oil–water) was employed for the formation of bFGF-encapsulated NPs. Briefly, 20 g of PLGA was dissolved in an appropriate amount of dichloromethane. The solution was then injected into the inner aqueous phase (W1, 100 μl of phosphate-buffer saline (PBS)) containing bFGF. The bFGF-W1 was emulsified with the polymer solution and the formation of small polymer droplets was facilitated by a high-speed IKA Ultra Turrax homogenizer (IKA, Guangzhou, China) operating at 225 × *g* for 2 minutes. The resulting emulsion (W1/O) was added to a larger aqueous phase (W2). A multiple emulsion (W1/O/W2) was achieved by homogenization with a high-speed homogenizer at the selected speed and time. After the complete evaporation of organic solvent with an evaporator, the copolymer was precipitated and formed solid NPs, which were collected by centrifugation. NPs were frozen in liquid nitrogen prior to lyophilization for freeze-drying.

### Analysis of BMSC surface markers by flow cytometry

The expressions of cell surface markers on BMSCs were evaluated by flow cytometry. BMSCs were harvested and stained with fluorescein isothiocyanate-conjugated mouse anti-rat CD44, CD73, CD90, and CD45, and affinity-purified mouse IgG1 as isotype control. Fluorescence-activated cell sorting was performed with FACSDiva (Canto, BD Bioscience, San Jose, CA, USA) and data were analyzed with FlowJo software (Tree Star, Ashland, OR, USA).

### 3-(4,5-Dimethyl-2-thiazolyl)-2,5-diphenyl-2-H-tetrazolium bromide assay

BMSCs were seeded at 10,000 cells per well in six-well plates and were incubated in 5 % carbon dioxide at 37 °C. At assay-specific time points, culture medium was replaced and wells were washed twice with PBS. In the growth curve experiment, 10 μl of 3-(4,5-Dimethyl-2-thiazolyl)-2,5-diphenyl-2-H-tetrazolium bromide (MTT, 0.5 mg/ml) was added and the culture was incubated for 4 hours. The culture medium was discarded and was replaced with 100 μl isopropanol/HCl. Absorbance at 570 nm was measured using a microplate reader. Relative viability was calculated using the following formula:

$$ \mathrm{Relative}\ \mathrm{viability} = \left(\mathrm{experimental}\ \mathrm{absorbance}\ \hbox{--}\ \mathrm{background}\ \mathrm{absorbance}\right)/\left(\mathrm{untreated}\ \mathrm{controls}'\ \mathrm{absorbance}\ \hbox{--}\ \mathrm{background}\ \mathrm{absorbance}\right)\kern0.5em  \times 100\ \%. $$

### Reverse transcription-PCR

Total RNA was extracted from cells using the RNeasy Plus Mini Kit (Qiagen, Gaithersburg, MD, USA). The cDNA was synthesized with the QuantiTect Reverse Transcription Kit (Qiagen) according to the manufacturer’s instructions. Expression levels were normalized to GAPDH. PCR was performed using the following primers: elastin, forward 5′-CTT CCT GGT GGA GTT CCC GGT GGA-3′, reverse 5′-CCG ATG CCA CCA ATA CCA CCG ACA-3′; collagen, forward 5′-GGA GAG TAC TGG ATC GAC CCT-3′, reverse 5′-CTG ACC TGT CTC CAT GTT GCA-3′; and GAPDH, forward 5′-ACC ACA GTC CAT GCC ATC AC-3′, reverse 5′-TCC ACC ACC CTG TTG CTG TA-3′.

### Western blotting

Cell or tissue samples were extracted with RIPA buffer (50 mM Tris–HCl, pH 7.4, 150 mM NaCl, 1 mM ethylenediamine tetraacetic acid, 1 % Triton X-100, 1 % sodium deoxycholate, 0.1 % sodium dodecyl sulfate, supplemented with protease inhibitors), and the total protein content was measured using the bicinchoninic acid protein assay kit (Thermo Scientific, Waltham, MA, USA). Proteins were resolved using denaturing SDS-PAGE followed by transferring onto nitrocellulose membrane. Primary antibodies against elastin (sc-17581), collagen (sc-8784), and actin (sc-8432) were purchased from Santa Cruz Biotechnology (Santa Cruz, CA, USA). Actin was used as the loading control. The immunoblot signal was detected using SuperSignal West Pico substrate (Pierce, Rockford, IL, USA) according to the manufacturer’s instructions.

### Enzyme-linked immunosorbent assay

Measurement of rat elastin and collagen was performed by enzyme-linked immunosorbent assay (ELISA) kits (Abbexa, Cambridge, UK) according to the manufacturer’s instructions. Briefly, collagen and elastin in samples was captured by the specific primary antibody and detected by the biotin-labeled secondary antibody. The assays were developed by avidin-peroxidase and its substrate, and plates were read at 450 nm using a microplate reader.

### Rat PFD model

The rat vaginal distention translational model with modification was set up in the study [31]. Briefly, an 18 F catheter was inserted into the rat vagina and then fixed with a single 3–0 silk suture. The Foley balloon was inflated with water (2.5–3.0 ml) and connected to a pressure transducer (about 0.15 kg) to create pressure on the pelvic floor support tissue. After 4 hours, the catheter was deflated and removed along with the pressure transducer from the vagina. Fourteen days after vaginal distention the LPP and CMG were assayed to ensure the establishment of the PFD model, and later for the evaluation of treatment outcome.

### Conscious cytometry

Two days before CMG, a bladder catheter (PE-50 tubing with a flared tip) was inserted into the PFD rat. The catheter was connected to a syringe pump (KD Scientific, New Hope, PA, USA) and a pressure transducer (Grass Instruments, West Warwick, RI, USA). Each bladder was filled with saline via the catheter at 5 ml/hour. A voiding contraction was defined as an increase of bladder pressure that resulted in urine loss as detected by a force transducer (Grass Instruments) that was calibrated to measure volume. Three fills and voids were recorded for each rat. Mean bladder baseline pressure, mean voided volume, mean peak voiding pressure, and mean increase in bladder pressure for voiding (peak voiding pressure minus bladder baseline pressure) were calculated for each animal with a chart recorder.

### LPP test

Two days before the LPP test, a bladder catheter (PE-50 tubing with a flared tip) was inserted into the PFD rat and connected via a stopcock to the pressure transducer and flow pump. Under anesthesia with urethane (1.2 g/kg body wt intraperitoneally), the bladder was palpated to empty and filled with saline at 5 ml/hour via the flow pump. When 0.3 ml was attained (approximately half the capacity of a 200 g rat), gentle pressure was applied with one finger to the rat’s abdomen to increase bladder pressure while bladder pressure was recorded and digitized. Pressure was slowly increased until the rat leaked saline through the urethra. At the first indication of leakage at the urethral meatus, the externally applied abdominal pressure was removed rapidly. Peak pressure at leakage in the absence of a detrusor contraction was recorded. The LPP was calculated by subtracting the bladder baseline pressure from the peak bladder pressure. The bladder was drained and refilled, and the study was repeated three times for each rat. Mean bladder baseline pressure and mean LPP were calculated for each rat.

### Statistical analysis

Statistical analyses were performed using SPSS software (SPSS Inc., Chicago, IL, USA). Results are shown as the mean ± standard deviation (SD). A two-group comparison was performed using the unpaired Student *t* test, and *P* <0.05 was considered statistically significant.

## Results

### Characterization of elastin-expressing BMSCs

**Fig. 1:**
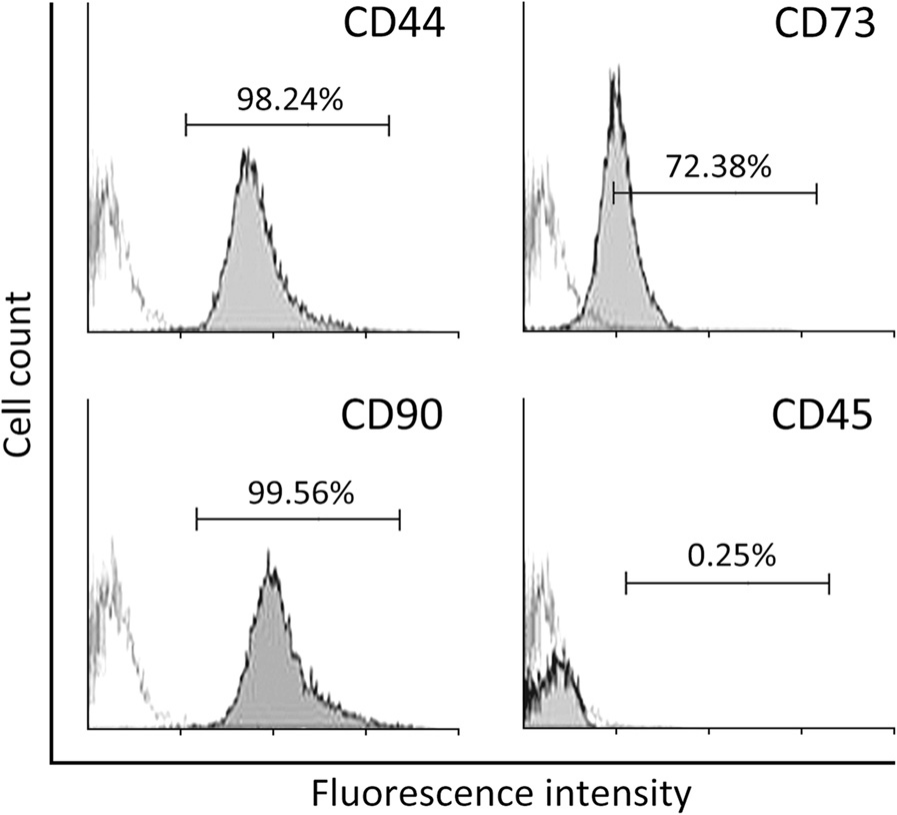
Flow cytometry identified rat BMSC-positive markers CD44, CD73, and CD90, but not negative marker CD45, on the surface of the cells

There are two kinds of stem cells in the bone marrow: mesenchymal and hematopoietic stem cells. To initiate the characterization of BMSCs, expression of the cell surface markers specific for mesenchymal and hematopoietic stem cells was analyzed by flow cytometry. All analyzed cells were positive for mesenchymal cell markers CD44, CD73, and CD90, but negative for hematopoietic cell marker CD45 (Fig. 1), confirming the identity of isolated BMSCs.

**Fig. 2:**
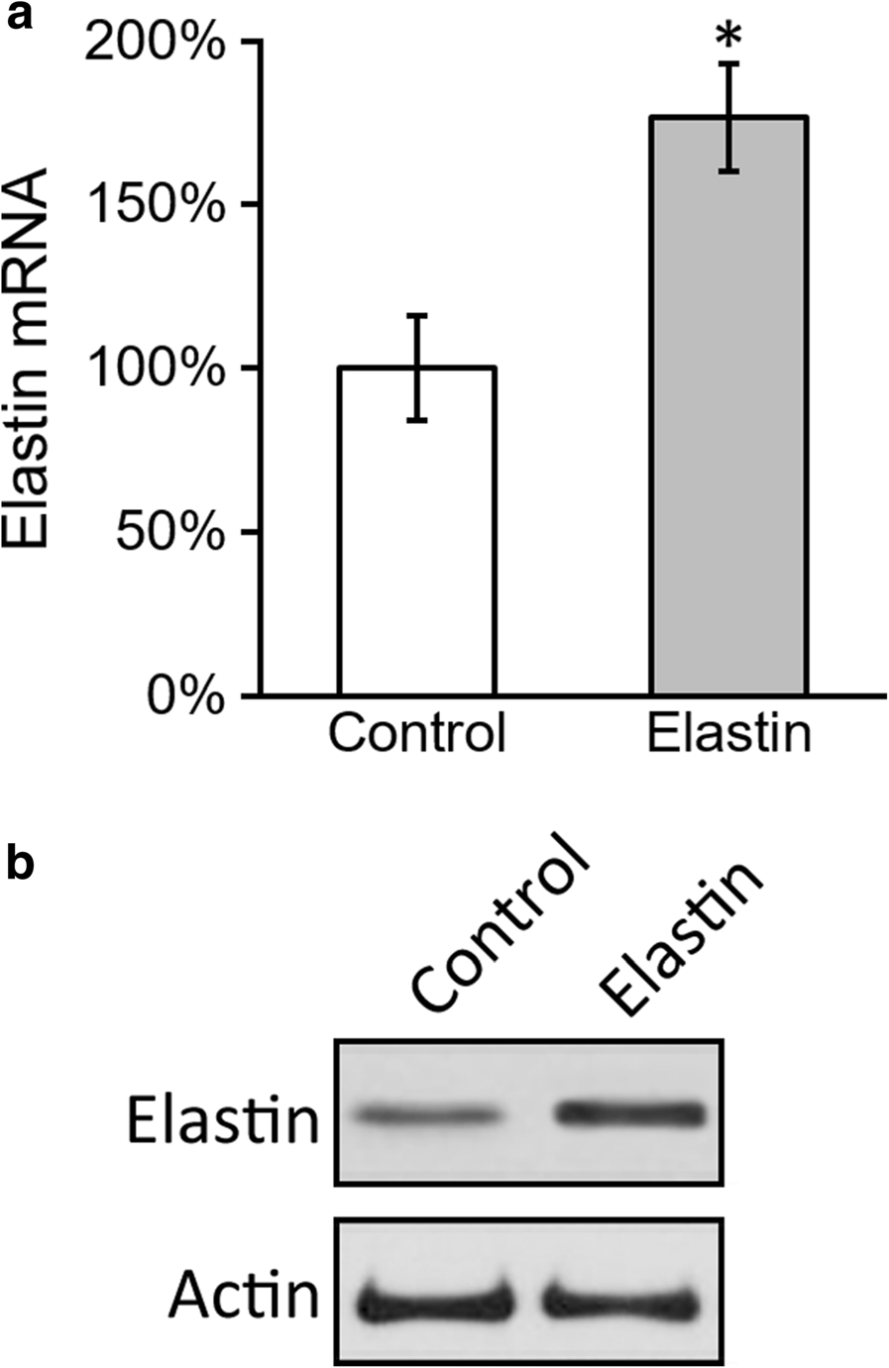
Effect of elastin transduction in BMSCs. Levels of mRNA **a** and protein **b** of elastin in BMSCs were examined 24 hours after transduction. Values are mean ± SD. *P <0.05 vs control

Next to confirm expression of the *elastin* gene, BMSCs infected with Ad-CMV-elastin-GFP were harvested 24 hours after infection. PCR and western blotting were performed to check the mRNA and protein expressions of elastin. Compared with BMSCs infected with empty vector Ad-CMV-GFP, BMSCs infected with Ad-CMV-elastin-GFP expressed significantly elevated elastin, both at mRNA and protein levels (Fig. 2).

### bFGF-loaded PLGA NPs promoted the proliferation of elastin-expressing BMSCs in vitro

**Fig. 3:**
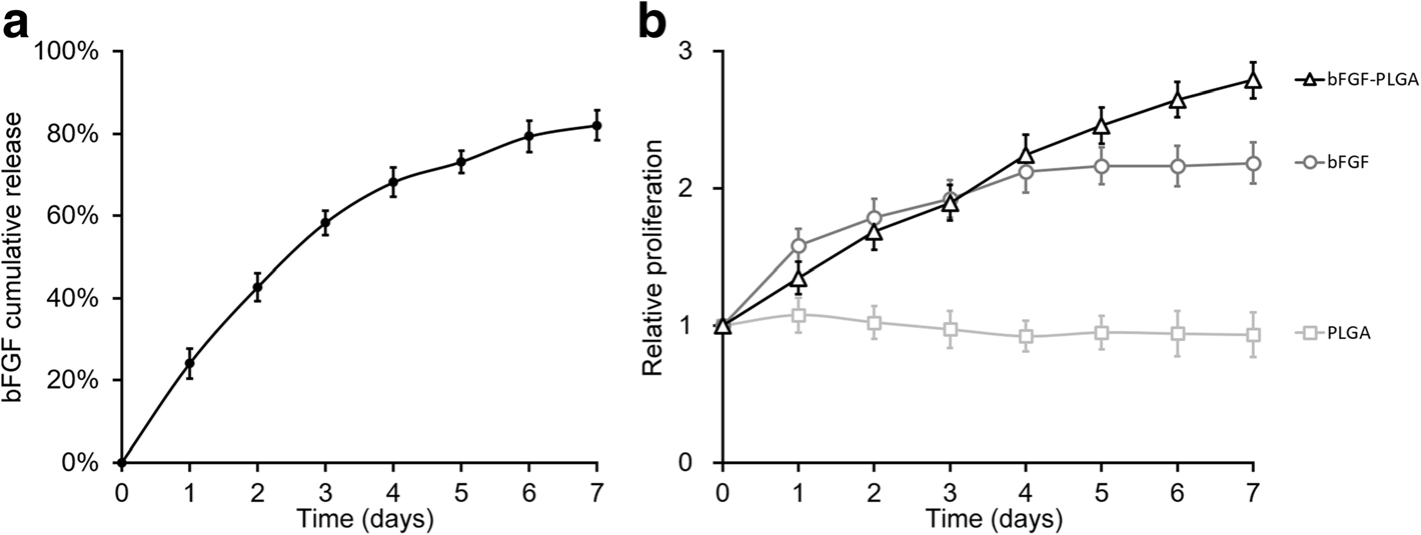
bFGF-PLGA promotes the proliferation of BMSCs. **a** In vitro cumulative release profile of bFGF-loaded PLGA. **b** Elastin-transfected BMSCs were cultured in the presence of either empty PLGA, free bFGF without PLGA (bFGF), or bFGF-loaded PLGA (bFGF-PLGA), and cell proliferation was measured by MTT assay. *bFGF* basic fibroblast growth factor, *PLGA* poly(lactic-co-glycolic acid)

The NP formulation provided a sustained bFGF release, which could serve as a reliable source of bFGF to promote the proliferation of BMSCs. We first measured the bFGF release curve in vitro. bFGF-loaded PLGA NPs (equivalent concentration 20 ng/ml) were incubated in medium for 7 days. The medium was harvested every day to assay for the release of bFGF using ELISA. As shown in Fig. 3a, approximately 70 % of bFGF was released from the NPs within the first 5 days in a consistent and sustained pace. The rest of the bFGF continued to be released until the end of the experiment at day 7.

Next, the bioactivity of the bFGF released from the PLGA NPs was evaluated via the induction capacity of BMSC proliferation. Elastin-expressing BMSCs were cocultured for 7 days with bFGF-loaded PLGA NPs at equivalent concentration 20 ng/ml (Fig. 3b, bFGF-PLGA), free bFGF at 20 ng/ml (Fig. 3b, bFGF), and empty PGLA as negative control (Fig. 3b, PLGA). No additional bFGF was supplemented in the culture (see Discussion). Cells were harvested daily for 7 consecutive days to measure proliferation using the MTT assay. In the empty PLGA NP control group, in the absence of bFGF, cells did not proliferate. Cells incubated with free bFGF proliferated in the first 4 days and the culture nearly doubled at day 4. However, cells stopped proliferating in the absence of replenishing bFGF, reaching the plateau after day 5. In contrast, cells incubated with bFGF-loaded PLGA proliferated as fast as the free bFGF group in the first 4 days. More importantly, with the sustained supply of bFGF released from the bFGF-loaded PLGA NPs, cells proliferated continuously with prolonged incubation time even after day 5. Taken together, the bFGF-loaded PLGA NP system proved to be an ideal sustained release system to be used for further experiments in our current study.

### bFGF-loaded PLGA promoted the expression and secretion of elastin and collagen in differentiated BMSCs in vitro

**Fig. 4:**
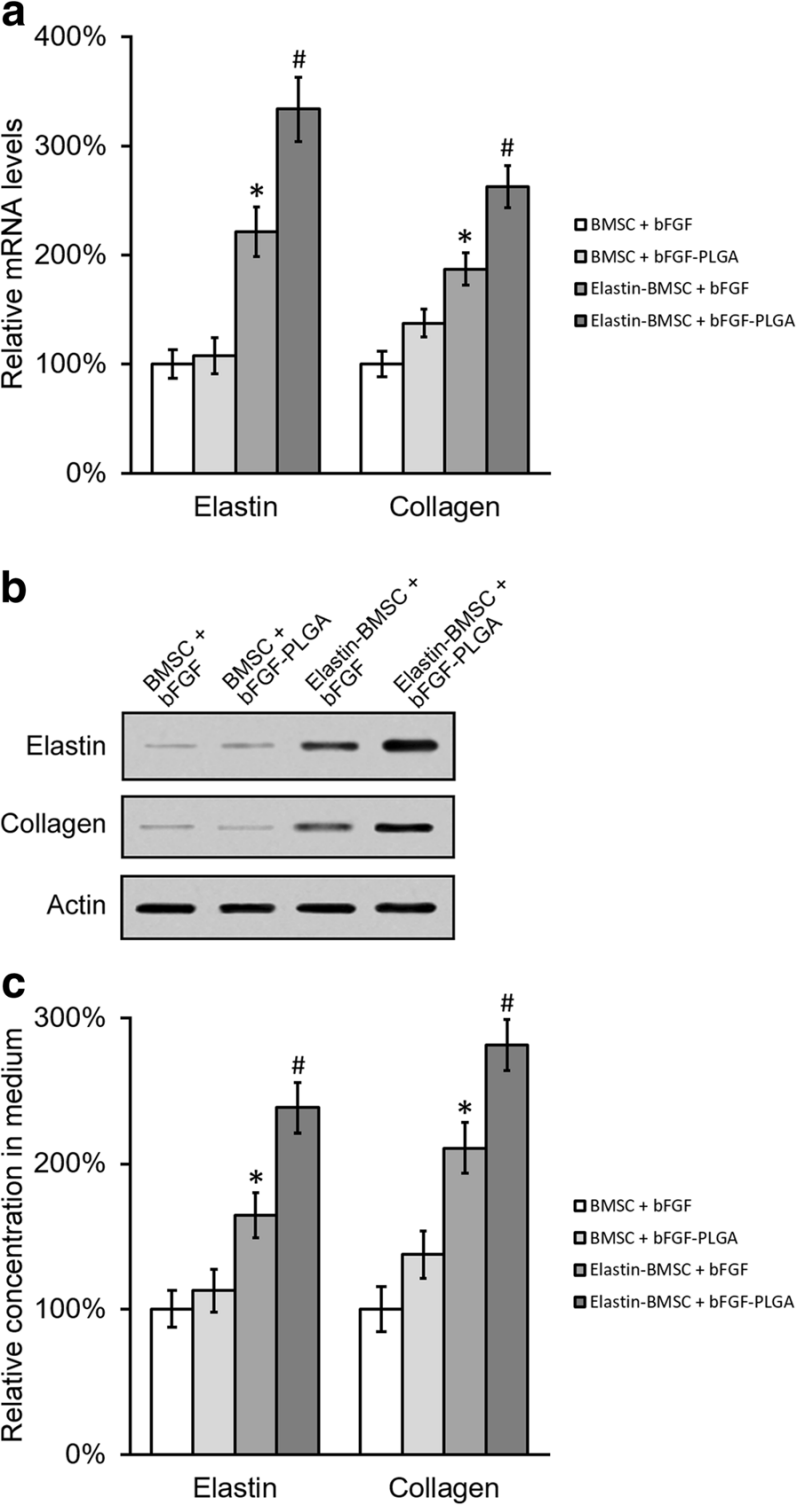
bFGF-PLGA promotes the expression and secretion of elastin and collagen in differentiated BMSCs. BMSCs with or without elastin transduction were differentiated in the presence of either bFGF or bFGF-PLGA for 7 days. Levels of mRNA **a** and protein **b**, and concentration in medium **c** of elastin and collagen were examined by RT-PCR, western blot, and ELISA assays. Values are mean ± SD. *P <0.05 vs BMSC + bFGF and BMSC + bFGF-PLGA. #*P* <0.05 vs elastin-BMSC + bFGF. *bFGF* basic fibroblast growth factor, *BMSC* bone marrow-derived mesenchymal stem cell, *PLGA* poly(lactic-co-glycolic acid)

The induction capacity of bFGF on the expression and secretion of elastin and collagen was investigated in differentiated BMSCs. Four different experimental groups were established: BMSC + bFGF, naked BMSCs cultured with free bFGF at 20 ng/ml; BMSC + bFGF-PLGA, naked BMSCs cultured with bFGF-loaded PLGA NPs at an equivalent concentration of 20 ng/ml; elastin-BMSC + bFGF, elastin-expressing BMSCs cultured with free bFGF at 20 ng/ml; and elastin-BMSC + bFGF-PLGA, elastin-expressing BMSCs cultured with bFGF-loaded PLGA NPs at an equivalent concentration of 20 ng/ml. Cells in all four groups were cultured in proliferation medium for 7 days and then changed to differentiation medium for a further 7 days. At the end of the experiment, cells from each group were harvested for evaluation of intracellular elastin and collagen expression using reverse transcription (RT)-PCR and western blotting, and supernatants of cell culture were also collected to measure the secretion of elastin and collagen into culture media using ELISA. In both RT-PCR and western blotting analysis, significantly increased expressions of elastin and collagen were observed in both groups of elastin-expressing BMSCs, compared with those from BMSCs not expressing elastin, regardless of incubation with either free bFGF or bFGF-loaded PLGA (Fig. 4a, b). In addition, administration of bFGF-loaded PLGA NPs to the elastin-expressing BMSCs further upregulated the expression of elastin and collagen, compared with the same cells incubated with free bFGF. Consistently, coculture of elastin-expressing BMSCs with bFGF-loaded PLGA exhibited a higher extent of stimulation on the secretion of elastin and collagen, compared with the same cells cocultured with free bFGF (Fig. 4c). These results indicated that overexpressing elastin in the BMSCs not only upregulated the expression of elastin itself, but also stimulated the intracellular production of collagen as well as extracellular secretion of both elastin and collagen.

### Transplantation of elastin-expressing BMSCs improved urodynamic testing in the rat PFD model

We next tested whether the elastin-expressing BMSCs could alleviate PFD symptoms in vivo, by establishing a rat PFD model. Six-month-old female Sprague Dawley rats were randomly and evenly divided into six experimental groups (*n* = 8 each): Control rats, normal rats without any intervention served as healthy controls throughout the duration of the experiment; PFD rats, saline was injected into the weakest position of the pelvis [28] 14 days after vaginal distention was performed on the rats to induce PFD symptoms (see Methods); BMSC + bFGF rats, naked BMSCs and free bFGF were coinjected into PFD rats 14 days after operation; BMSC + PLGA-bFGF rats, naked BMSCs and bFGF-loaded PLGA were coinjected into PFD rats 14 days after operation; elastin-BMSC + bFGF rats, elastin-expressing BMSCs and free bFGF were coinjected into PFD rats 14 days after operation; and elastin-BMSC + PLGA-bFGF rats, elastin-expressing BMSCs and bFGF-loaded PLGA were coinjected into PFD rats 14 days after operation. Seven days after injection, the two urodynamic tests of CMG and LPP were performed in all of these experimental groups of rats.

**Fig. 5:**
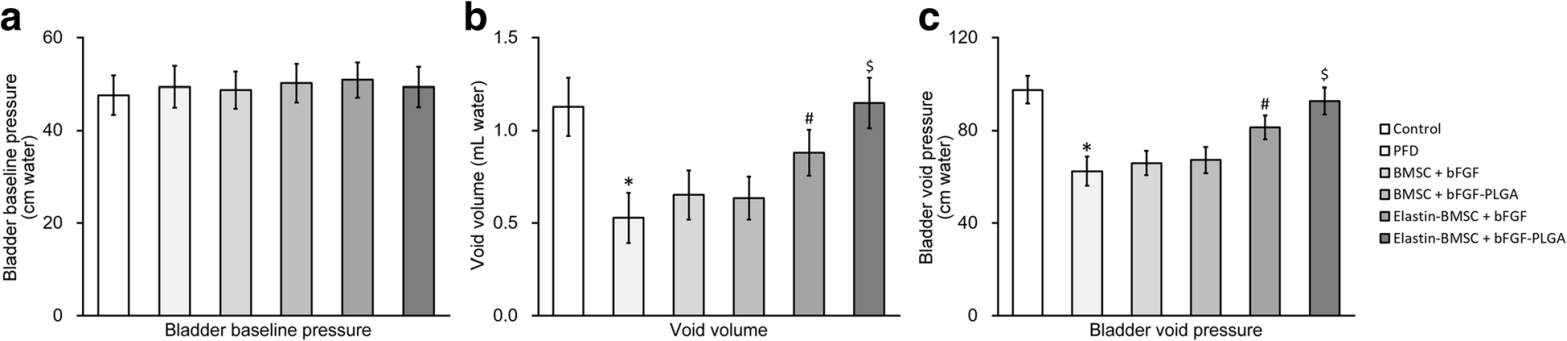
bFGF-PLGA and elastin transfection improve CMG in PFD rats after BMSC transplantation. The rat PFD model was established and housed for 14 days, after which the rats were transplanted with BMSCs and housed for a further 7 days. CMG including **a** bladder baseline pressure, **b** void volume, and **c** bladder void pressure was then performed on all rats (*n* = 8) in each experimental groups. Values are mean ± SD. *P <0.05 vs Control. #*P* <0.05 vs PFD. $*P* <0.05 vs Control and elastin-BMSC + bFGF. *bFGF* basic fibroblast growth factor, *BMSC* bone marrow-derived mesenchymal stem cell, *PFD* pelvic floor dysfunction, *PLGA* poly(lactic-co-glycolic acid)

In the CMG test, similar levels of bladder baseline pressure were observed in all experimental groups of rats (Fig. 5a). As expected, the void volume and bladder void pressure were significantly decreased in the PFD rats compared with the Control rats (Fig. 5b, c, first and second columns), suggesting the successful establishment of a rat PFD model. Coinjection of naked BMSCs along with either free bFGF or bFGF-loaded PLGA did not reverse the decreased void volume or bladder void pressure (Fig. 5b, c, third and fourth columns). In contrast, both the decreased void volume and bladder void pressure were reversed in rats receiving injections of elastin-expressing BMSCs (Fig. 5b, c, fifth and sixth columns). More importantly, aided by the sustained release of bFGF from PLGA NPs, the void volume and bladder void pressure of elastin-BMSC + bFGF-PLGA rats were almost completely restored to the same level as the Control rats (Fig. 5b, c, sixth column).

**Fig. 6:**
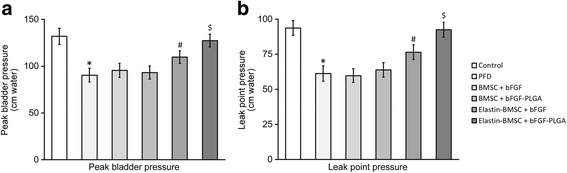
bFGF-PLGA and elastin transfection improve LPP in PFD rats after BMSC transplantation. The PFD rat model was established and housed for 14 days, after which the rats were transplanted with BMSCs and housed for a further 7 days. **a** Peak bladder pressure and **b** LPP were then performed on all rats (*n* = 8) in each experimental group. Values are mean ± SD. *P <0.05 vs Control. #*P* <0.05 vs PFD. $*P* <0.05 vs Control and elastin-BMSC + bFGF. *bFGF* basic fibroblast growth factor, *BMSC* bone marrow-derived mesenchymal stem cell, *PFD* pelvic floor dysfunction, *PLGA* poly(lactic-co-glycolic acid)

In LPP testing, the peak bladder pressure and LPP were also significantly decreased in PFD rats compared with the Control rats (Fig. 6a, b, first and second columns), which further demonstrated the successful establishment of a rat PFD model in our current study. Expectedly, coinjection of naked BMSCs along with either free bFGF or bFGF-loaded PLGA had minimal impact on peak bladder pressure and LPP (Fig. 6a, b, third and fourth columns), compared with PFD rats. Injection with elastin-expressing BMSCs reversed the decreased void volume and bladder void pressure (Fig. 6a, b, fifth and sixth columns). Furthermore, when coinjected with bFGF-loaded PLGA, elastin-expressing BMSCs rescued the decreased peak bladder pressure and LPP to a level indistinguishable from those of Control rats (Fig. 6a, b, sixth column).

In summary, gene modification of BMSCs without elastin had minimal effect on the outcomes of urodynamic tests in PFD rats, regardless of the source of bFGF. Together with the sustained release of bFGF from PLGA NPs, however, elastin-expressing BMSCs significantly improved the outcome of urodynamic tests to levels similar to those of healthy rats.

## Discussion

Weakening of pelvic connective tissues is considered a contributing factor in PFD. Elastin is a critical component of vaginal and pelvic floor connective tissues. Increasing numbers of studies have begun to focus on the role of elastin in the pathophysiological development of PFD, especially in SUI and POP, both of which are associated with childbirth injuries. During delivery, distention of the vagina results in the degradation of elastin fibers. Destructed elastin fibers are improperly organized, leading to malfunction which eventually causes tissue stiffness. Studies in a mouse model of defective elastin have suggested that elastic fiber synthesis is a critical process of childbirth recovery, and impaired elastin synthesis leads to prolapse [32]. The prevalence of PFD is high in women and current treatment options are suboptimal.

Given that altered elastin metabolism and connective tissue abnormality represent the major pathophysiological development of PFD, our study focused on an elastin gene-modified, stem-cell based, and NP-mediated multidisciplinary therapy for the repair and regeneration of pelvic floor tissues in PFD. To alter the matrix composition and change the properties of the damaged stiff tissue, we introduced the *elastin* gene into BMSCs which were then transplanted into the vaginal distension-induced damaged tissue in adult rats. We demonstrated that bFGF-loaded PLGA NPs could markedly stimulate the secretion of collagen and elastin, following differentiation of elastin-expressing BMSCs to fibroblasts. Transplantation of elastin-expressing BMSCs in vivo yielded favorable therapeutic effects on PFD rats, where bFGF-loaded PLGA provided sustained release of bFGF and further enhanced the beneficial effects of elastin-expressing BMSCs.

Mesenchymal stem cells have already exhibited great potential in soft tissue reconstruction [33, 34]. In a recent in vivo study on PFD [35], transplantation of BMSCs resulted in new tissue growth and collagen deposit in a wound-healing model. In the context of PFD, to functionally restore pelvic floor support, an appropriate amount of elastic fibers in the connective tissue is extremely important. Simple deposition of collagen would cause formation of dense connective tissues and eventually scar tissues. The potential solution is elastin gene-engineered cell therapy [27], where overexpressing elastin in BMSCs has led to restoration of elasticity in the dilated cardiac tissue and recovery of cardiac function. Consistent with previous study [27], elastin-expressing BMSCs produced sufficient elastin and collagen in our rat PFD model to restore the function of tissues damaged by virginal distention.

Fibroblasts in the vaginal wall were sensitive to the mechanical stretch during delivery [36]. When cultured with mechanical stretching, fibroblasts isolated from the vaginal wall in women suffering from POP grew perpendicular to the direction of the force, and exhibited decreased cell projection area and cell circularity, and increased cell length/width ratio, suggesting that POP fibroblasts have greater sensitivity and less tolerance for mechanical stretching [37]. Repeated mechanical stretching eventually leads to a degradation response of fibroblasts. Theoretically, recovery in the quantity and function of fibroblasts should be beneficial for tissue repair, and therefore replacement of the damaged fibroblasts at the injury site using mesenchymal stem cell therapy could be beneficial. In our study, elastin-expressing BMSCs supplemented with bFGF produced sufficient elastin and collagen in vitro, and exhibited significant beneficial effects on PFD rats in vivo, although the exact mechanism underlying the improved clinical outcome was not well defined because the development of mesenchymal stem cell transplantation therapy is still in the early stage.

Although the fate of transplanted BMSCs after injection is unclear, limited long-term engraftment is commonly seen [38]. No prelabeled stem cells were found up to 14 days in the injection site, even though the bioactivity of the stem cells can last much longer, with a possible explanation being that the stem cells undergo proliferation and differentiation quickly. Nevertheless, controlling the fate and the phenotype of engrafted BMSCs in tissues remains a big challenge. Unlike in vitro cell culture, where growth factors such as bFGF used in our current study can be resupplied easily and readily, growth factors distribute quickly inside the tissue in the bioenvironmental milieu and degrade rapidly. Replenishing growth factors in vivo is challenging, even though repeated high-dose injection could be an alternative albeit expensive way. Besides, burst doses, inconsistent local concentration, high cost, and injection-associated complications are obvious disadvantages. Moreover, the lack of growth factors in transplanted stem cells leads to variability in cell function and ultimately poor therapeutic outcomes.

PLGA provides a platform to control the local pharmacokinetics of growth factors, and in turn the proliferation and differentiation of transplanted stem cells. As an FDA-approved drug delivery device, PLGA NPs can be easily processed and fabricated in various forms and sizes, and have many attractive properties, such as biocompatibility and biodegradability. In our current study, PLGA was used to control the sustained release of bFGF. Comparing the elastin-expressing BMSCs cocultured with either free bFGF or bFGF-loaded PLGA, the latter experimental group exhibited enhanced expression and secretion of both collagen and elastin in vitro. In the rat PFD model, bFGF-loaded PLGA along with elastin-expressing BMSCs significantly alleviated the PFD symptoms. We speculated that delivery of the growth factor in a controlled and sustained manner could maintain the therapeutic effects of transplanted BMSCs over time and effectively direct the differentiation of stem cells, where their ability to secret elastin and collagen was subsequently improved. In this context, our current report may provide a proof-of-principle study that the combinational stem cell and NP therapy would be useful and very interesting to apply in future PFD-related investigations, particularly the correlation between the prolapse stage and proliferation after 7 days, as well as the mechanical properties of tissues in the PFD animal model.

## Conclusion

We have demonstrated in the first instance that transplantation of elastin-expressing BMSCs into PFD rats could significantly alleviate the symptoms of PFD. This beneficial effect could be further enhanced by sustained released of bFGF from PLGA NPs. By taking a multidisciplinary approach combining genetic modification, stem cell therapy, and NP techniques, our current study presented an efficient and promising method using genetically modified BMSCs to promote the reconstitution of connective tissue defects and to treat PFD.

## Abbreviations

Ad: Adenovirus
bFGF: Basic fibroblast growth factor
BMSC: Bone marrow-derived mesenchymal stem cell
CMG: Conscious cytometry
CMV: Cytomegalovirus
ECM: Extracellular matrix
ELISA: Enzyme-linked immunosorbent assay
FDA: Food and Drug Administration
GFP: Green fluorescent protein
LPP: Leak point pressure
MTT: 3-(4,5-Dimethyl-2-thiazolyl)-2,5-diphenyl-2-H-tetrazolium bromide
NP: Nanoparticle
PBS: Phosphate-buffer saline
PFD: Pelvic floor dysfunction
PLGA: Poly(lactic-co-glycolic acid)
POP: Pelvic organ prolapse
RT: Reverse transcription
SD: Standard deviation
SUI: Stress urinary incontinence
